# Differential compartment overdrainage syndrome

**DOI:** 10.1186/2045-8118-12-S1-P9

**Published:** 2015-09-18

**Authors:** Claudia Craven, Neekhil A Patel, Samir A Matloob, Edward W Dyson, Aswin Chari, Tarek Mostafa, Simon D Thompson, Patricia Haylock-Vize, Syed N Shah, Andrew R Stevens, Huan Wee Chan, Jinendra Ekanayake, Ahmed K Toma, Laurence D Watkins

**Affiliations:** 1 Victor Horsley Department of Neurosurgery, National Hospital for Neurology and Neurosurgery, Queen Square, London, UK

## Introduction

We describe a consistently similar clinical presentation of patients with complex hydrocephalus and encysted fourth ventricle separately drained by infratentorial shunt insertion.

## Methods

A retrospective single centre case series. Medical notes were reviewed for clinical presentation, brain imaging and neurophysiological tests results. All patients underwent ICP monitoring using Spiegelberg bolt. Outcomes were determined by retrospective analysis of 24-hour ICP monitoring results, ventricular appearance on brain imaging CT and symptomatic improvements post-operatively.

## Results

Five adult patients were referred to the hydrocephalus service in our unit with separate infra and supratentorial shunt systems. Clinical presentation included: bilateral lower motor neuron facial palsy, ophthalmoplegia, dysphonia, impaired gait headache and nausea. No patient experienced deafness. Two subjects had their facial nerve palsy confirmed with electrophysiology studies. Brain stem evoked potentials also confirmed sparing of the 8th cranial nerve. 24 hours ICP monitoring confirmed clear low pressures.

These patients underwent shunt revision connecting the supra and infratentorial shunt systems to achieve equal pressure drainage, with subsequent addition of a distal valve. Further ICP monitoring confirmed normalisation of pressure. All patients reported improvement in headaches and nausea, with a mild improvement in gait and dysphagia. On the other hand, facial weakness and opthalmoplegia were persistent. All subjects had improved ventricular appearance on CT scans post revision. In the follow up period of 3 months no patient required further shunt revision.

## Conclusion

Supra and infratentorial shunt construct in adults with encysted fourth ventricles should be similar to the shunt system widely known in the paediatric population with Dandy Walker syndrome, i.e. joint output to a single valve distal to the connection of the 2 drainage proximal catheters.

